# Exercise and resveratrol increase fracture resistance in the 3xTg-AD mouse model of Alzheimer’s disease

**DOI:** 10.1186/s12906-019-2451-6

**Published:** 2019-02-04

**Authors:** Mustafa F. Alkhouli, Jun Hung, Michaela Squire, Miranda Anderson, Monica Castro, Jeganathan R. Babu, Layla Al-Nakkash, Tom L. Broderick, Jeffrey H. Plochocki

**Affiliations:** 1 Arizona College of Osteopathic Medicine, Midwestern University, Glendale, AZ USA; 2 Department of Anatomy, Arizona College of Osteopathic Medicine, Midwestern University, Glendale, AZ USA; 3 Department of Nutrition, Dietetics, and Hospitality Management, Auburn University, Auburn, AL USA; 4 Department of Physiology, Arizona College of Osteopathic Medicine, Midwestern University, Glendale, AZ USA; 5 Department of Physiology, Laboratory of Diabetes and Exercise Metabolism, Arizona College of Osteopathic Medicine, Midwestern University, Glendale, AZ USA; 6 Department of Medical Education, University of Central Florida, College of Medicine, 6850 Lake Nona Blvd, Orlando, FL 85308 USA

**Keywords:** Alzheimer’s, Exercise, Resveratrol, Fracture

## Abstract

**Background:**

Alzheimer’s disease (AD) and osteoporosis are progressive diseases that affect the elderly population. Both conditions are associated with fracture risk that is greater than twice that of the healthy population. Resveratrol and exercise are two treatments that have been linked with attenuation of age-related diseases, including the risk of bone fractures. In this study, we test the hypothesis that these treatments improve fracture resistance in a mouse model representative of the AD condition.

**Methods:**

Three-month-old male 3xTg-AD mice were treated for 4 months with resveratrol or exercise or both combined, and compared with wild type mice. Exercise training was performed on a treadmill at 15 m/min for 45 min/day, 5 days/week. Resveratrol was given at 4 g/kg diet in the form of pellets. Three-point bending, cross-sectional geometric, and fluorescence analyses were conducted on tibias and compared by treatment group.

**Results:**

Tibias of 3xTg mice exhibited signs of diminished bone quality and fracture under less force than age-matched wild type mice (*P* < 0.05). Treatment with both resveratrol and exercise improved indicators of fracture resistance and bone quality in AD mice to levels comparable to that of wild type mice (*P* < 0.05).

**Conclusions:**

The 3xTg mouse model of AD is at elevated risk for limb bone fracture compared to wild type controls. Treatment with resveratrol, exercise, or both in combination improves fracture resistance and cross-sectional geometric indicators of bone strength.

## Background

Alzheimer’s disease (AD) is the fifth leading cause of death in the United States and is on the rise worldwide [1, 2]. AD is characterized by progressive cognitive decline associated with synaptic dysfunction and the accumulation of amyloid-beta (Aβ) plaques and neurofibrillary tangles in brain tissue [3]. Osteoporosis is another age-related disease that affects millions of adults around the world over the age of 65 [4]. Osteoporosis is a degenerative disease that results in the loss of bone mineral density and is associated with increased fracture risk and mortality [5]. AD and osteoporosis share risk factors and etiologies, and the incidence of either one increases the risk of developing the other [6–8]. When both diseases are present, population studies have shown an increased mortality risk in comparison to non-AD patients alone [9].

There is mounting evidence indicating that regular physical activity and a diet rich in polyphenols have the potential to impact a variety of age-related human diseases. Epidemiological and clinical studies indicate that exercise can prevent the onset or slow the progression of neurodegenerative and metabolic diseases, including AD and osteoporosis [10–12]. These studies also emphasize the importance of polyphenol-rich diets for the management of osteoporosis [13–15]. Resveratrol (3,5,4-trihydroxy-trans-stilbene) is a naturally occurring polyphenol found in relatively high concentrations in grapes, seeds, and nuts is known to exert significant bone-protective effects [16]. Specifically, dietary resveratrol activates antioxidative and osteoblastic proliferative pathways (SIRT1 and Fox01), increasing biochemical indicators of bone quality and stimulating osteogenesis to improve bone mineral density in osteoporotic animals [17–19]. Activation of the SIRT1 pathway by resveratrol also improves cognitive and memory deficits and is being investigated as a treatment for AD [20]. However, exercise and resveratrol treatment, either alone or in combination, have yet to be studied in regards to their potential ability to reduce AD-related fracture risk. In this study, we test the efficacy of resveratrol and/or exercise in ameliorating fracture risk in the 3xTg-AD mouse model for AD. In this study, we examine multiple indicators of bone strength and quality to determine their effects on resistance to limb bone fracture.

## Methods

### Experimental design

Three-month-old male triple-transgenic mice (3xTg-AD) harboring three mutant genes; amyloid-β precursor protein, presenilin-1, and tau, were purchased from Jackson Laboratories (Bar Harbor, ME, USA). Non-transgenic, aged-matched, wild type littermates (B6129SS2/J) served as a control. After a one-week period of acclimation, mice were randomly assigned to the following groups: control (*n* = 10), 3xTg-AD control (*n* = 7), 3xTg-AD treated with resveratrol (*n* = 8), 3xTg-AD with exercise (*n* = 8), and 3xTg-AD treated with a combination of resveratrol and exercise (n = 8). Duration of treatment was 4 months, after which mice were sacrificed by asphyxiation using compressed CO2 at a flow rate of 1 L/min and chamber fill rate of 30% chamber volume per minute. Throughout the experiment, mice were housed in an animal facility at a temperature of 22 °C and a 12-h light/dark cycle and given food and water ad libitum. Animal use was approved by the Institutional Animal Care and Use Committee at Midwestern University and adhered to the guidelines in the National Institutes of Health’s Guide for the Care and Use of Laboratory Animals.

Mice in the resveratrol groups were administered resveratrol (Lalilab Inc., Durham, NC, USA) in diet (4 g/kg, AIN-93G, Dyets Inc., Bethlehem, PA), while the control group received regular diet without resveratrol. The resveratrol dosage was selected based on previous studies showing this amount provides sufficient bioavailability to have an active effect with dietary administration in mice (equivalent to ∼146 mg kg^− 1^ day^− 1^) [21, 22]. In addition, this dosage exerts an insulin-mimetic effect, reduces oxidative stress, and activates the SIRT1 anti-apoptotic signaling pathway [22–24].

Exercise training consisted of forced running on a motor-driven treadmill designed for mice (Exer 3/6 treadmill; Columbus Instruments, Columbus, OH, USA). Mice were initially placed in separate lanes of the treadmill while it was off (0 m/min) and gradually acclimated to daily 10-min running sessions at 10 m/min for a period of one week. The duration and intensity were increased to 20 min at 10 m/min on week 2, followed by 30 min at 12 m/min on week 3. From week 4 to the end of the training regimen, running activity consisted of 45 min at 15 m/min, 5 day/week, corresponding to an estimated submaximal VO_2_ of ~ 50 mL/kg/min [25]. At this intensity, mice were running without reluctance and were able to continue for the entire 45-min session.

### Three-point bending test

Following sacrifice, tibias were harvested and cleaned of soft tissue. Tibial length and midshaft diameter were measured using digital calipers. Right tibias were subjected to three-point bending until failure to evaluate resistance to fracture as previously described [26]. A load was applied to the midshaft of the tibias using a round-edged tip in the anterior-posterior axis at a rate of .05 N/s (HP-5 Force Gauge and HSV Test Stand; Handpi Instruments Co., Ltd., China). Tibias were mounted on supports that were positioned to contact the proximal and distal ends. The span between the supports was varied such that they were located at the same relative distance along the length of the tibia. This enables standardization of the location of the loading point, because load-to-fracture is proportional to the distance between the supports and the diameter of the bone in the breaking plane. At the conclusion of the bending test, data on ultimate force, ultimate stress, maximum bending moment, stiffness, and elastic modulus were recorded. Ultimate force and ultimate stress are the maximum force and stress values recorded values during the test, and usually occurred around the time of fracture. Maximum bending moment is the largest rotational force that causes bending. Stiffness and elastic modulus are measures of the stress-strain relationship for a given material that reflects how it deforms under loading.

### Cross-sectional geometry

After sacrifice, left tibias were measured and a pencil mark was made at midshaft such that the midshaft could be identified during sectioning. Tibias were then dehydrated in graded alcohol and cleared (Histoclear, National Diagnostics, Atlanta, Georgia, USA) in two 24-h washes. Bone tissue was infiltrated with catalyzed Osteo-Bed Resin A for 2 days and then submerged in Osteo-bed resin (Polysciences Inc., Warrington, PA, USA). Polymerization of the resin was in a bead bath held at 33.5 °C for 4 days. Following hardening, an undecalcified transverse section of the tibial midshaft was made at a thickness of 100 μm using a low speed saw (Isomet; Buehler, Lake Bluff, IL, USA) and polished with a fine cloth pad (MetaServ; Buehler, Lake Bluff, IL, USA). Sections were imaged under bright field microscopy at 4X magnification. The MomentMacroJ plugin (M Warfel and S Serafin) for ImageJ v1.8 (NIH) was used to measure total area (Tt.Ar) cortical area (Ct.Ar), and medullary area (M.Ar) of the tibial midshaft, and to calculate maximum and minimum second moments of area (Imax and Imin) and polar moment of area (J). Ct.Ar, Imax and Imin, and J are cross-sectional properties that measure a long bone’s ability to resist compressive, bending, and torsional loads, respectively.

### Quantitative fluorescence

Undecalcified tibial sections were imaged with fluorescence microscopy to quantify fluorescence. Corrected total bone fluorescence (CTBF) was calculated in ImageJ v1.8 as the product of the cross-sectional area of the tibia and the mean gray value of the bone standardized to the mean background color. This formula is adapted from the method used to obtain total corrected cell fluorescence in intracellular immunofluorescence studies [27, 28]. Fluorescence in bone is a reflective of post-translational modification of structural proteins, primarily collagen type I, through nonenzymatic glycation [29]. The presence of such advanced glycation end-products (AGEs), which have been implicated in osteoporotic bone fractures, are positively correlated with increased fluorescence and are associated with increased fracture risk [30, 31].

### Statistical analysis

Statistical analyses of the data were conducted using SPSS Statistics 25 software (IBM, USA). Comparisons between wild type and 3xTg-AD mice were made using unpaired two-sample t tests. AD treatment groups were tested against AD controls to determine if treatment significantly affected each variable. Statistical significance was set at *P* < 0.05. Tests of power, normality, and homogeneity of variance were conducted to ensure there was sufficient statistical power, and to reduce the likelihood of type II errors and violations of statistical assumptions of normality.

## Results

No signs of distress or poor health were observed and all mice completed the exercise regimen during the study. Therefore, no modifications were made to the study design or research protocols during the experiment and all animals were included in all of the following analyses.

### Treatment with resveratrol and exercise improves fracture resistance

**Fig. 1:**
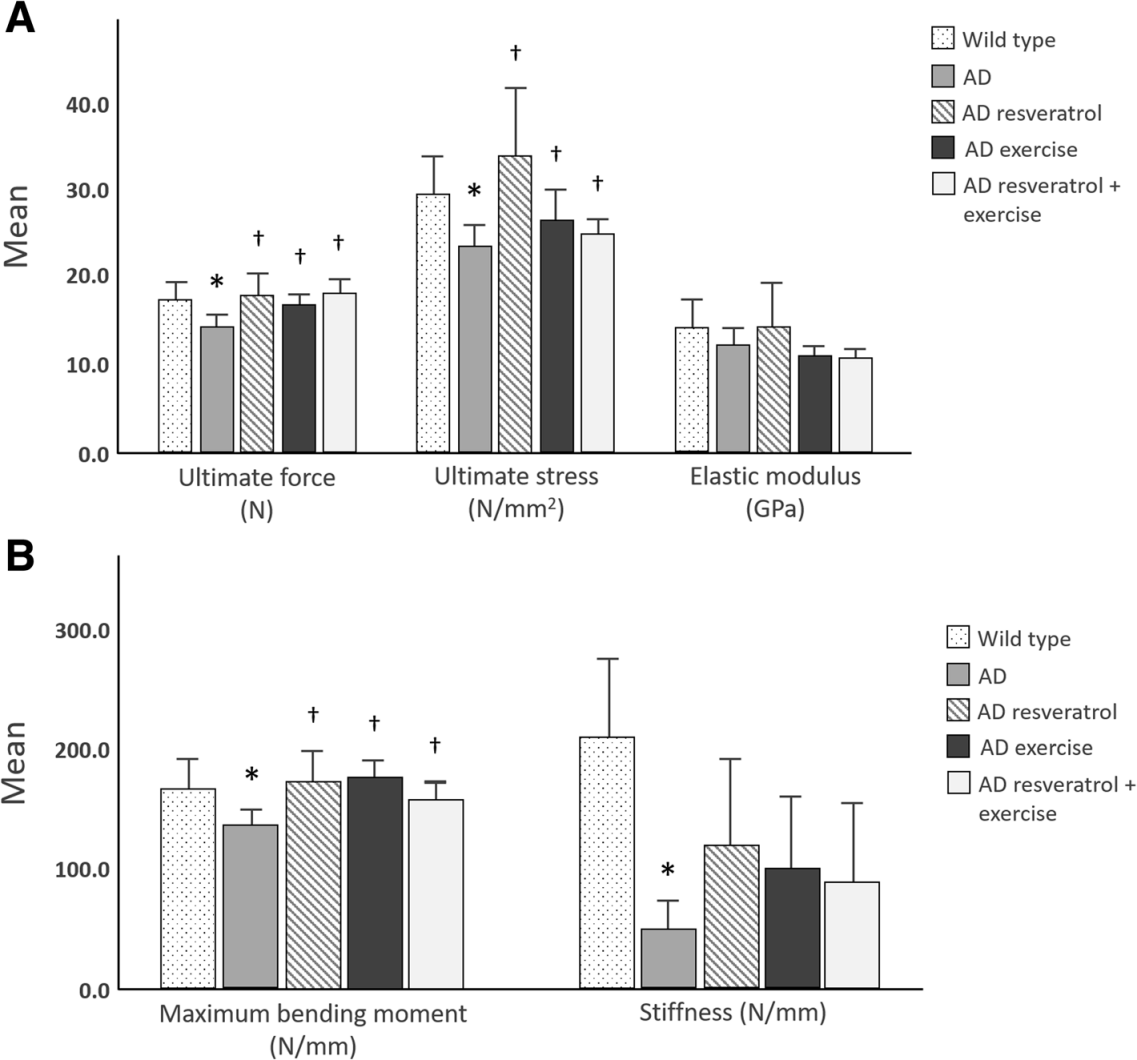
Results of three-point bending testing. **a** Tibias of AD control mice require less force and stress to fracture than wild type mice (*P < 0.05). Treatment with resveratrol and exercise increase ultimate force and ultimate stress (†*P* < 0.05), but not elastic modulus (*P* > 0.05) in comparison to AD controls. **b** Maximum bending moment was lowest in AD controls (*P < 0.05) and was reversed by resveratrol and exercise treatment (†*P* < 0.05). Tibias of AD control mice are less stiff than wild type mice *P < 0.05), but treatment did not affect tibial stiffness (†*P* > 0.05). Error bars are 2 ± SE

During three-point bending testing, tibias of AD control mice fractured under the least amount of force (Fig. 1). Ultimate force and ultimate stress were significantly lower for the tibias of AD controls than wild type mice (*P* < 0.05). Maximum bending moment and stiffness of AD control tibias were also significantly lower than that of wild type mice (*P* < 0.05). Treatment with resveratrol or exercise or both in combination increased the ultimate force, ultimate stress, and maximum bending moment in comparison to AD controls (*P* < 0.05). Elastic modulus did not differ between treatment groups.

### Treatment with resveratrol and exercise in combination increases resistance to loading

Analysis of cross-sectional geometric properties of the tibia midshaft of 3xTg-AD mice treated with both resveratrol and exercise exhibited the greatest resistance to compression, bending and torsion loading, even greater than that of wild type mice (Table 1, *P* < 0.05). 3xTg-AD mice treated with resveratrol and exercise in combination also exhibited greater tibial diameter and cross-sectional total area and cortical area than 3xTg-AD controls (*P* < 0.05). In comparison to wild type mice, 3xTg-AD mice had smaller medullary areas (*P* < 0.05), but were otherwise similar in their cross-sectional profile. Treatment with resveratrol alone or exercise alone did not result in improvements in load resistance.

**Table 1:** Cross-sectional geometric properties of the tibia midshaft

	Wild type	3xTg-AD control	3xTg-AD resveratrol	3xTg-AD exercise	3xTg-AD resveratrol + exercise
Length (mm)	19.5 ± 0.15	19.2 ± 0.13	19.1 ± 0.15	19.3 ± 0.17	19.3 ± 0.15
Diameter (mm)	0.69 ± 0.03	0.69 ± 0.02	0.69 ± 0.03	0.70 ± 0.05	0.76 ± 0.04*
Tt.Ar (mm^2^)	0.86 ± 0.04	0.78 ± 0.26	0.84 ± 0.03	0.83 ± 0.10	0.93 ± 0.03*
M.Ar (mm^2^)	0.27 ± 0.02	0.17 ± 0.01	0.19 ± 0.01	0.18 ± 0.02	0.20 ± 0.01
Ct.Ar (mm^2^)	0.59 ± 0.03	0.60 ± 0.02	0.64 ± 0.03	0.64 ± 0.07	0.74 ± 0.03*
Imax (×10^−2^, mm^4^)	7.51 ± 0.78	6.69 ± 0.37	7.34 ± 0.73	8.06 ± 1.45	9.58 ± 0.83*
Imin (× 10^− 2^, mm^4^)	4.33 ± 0.45	3.78 ± 0.33	4.68 ± 0.51	4.73 ± 0.78	5.65 ± 0.45*
J (×10^−2^, mm^4^)	11.8 ± 1.20 0.06	10.5 ± 0.69	12.0 ± 1.22	12.8 ± 2.22	15.2 ± 1.24*

Data displayed as mean ± SE; Tt.Ar, total area; M.Ar, medullary area; Ct.Ar, cortical area; Imax, maximum second moment of area; Imin, minimum second moment of area; J, polar moment of area. *, *P* < 0.05 in comparison with 3xTg-AD control

### 3xTg-AD mice have greater AGE content than wild type mice

**Fig. 2:**
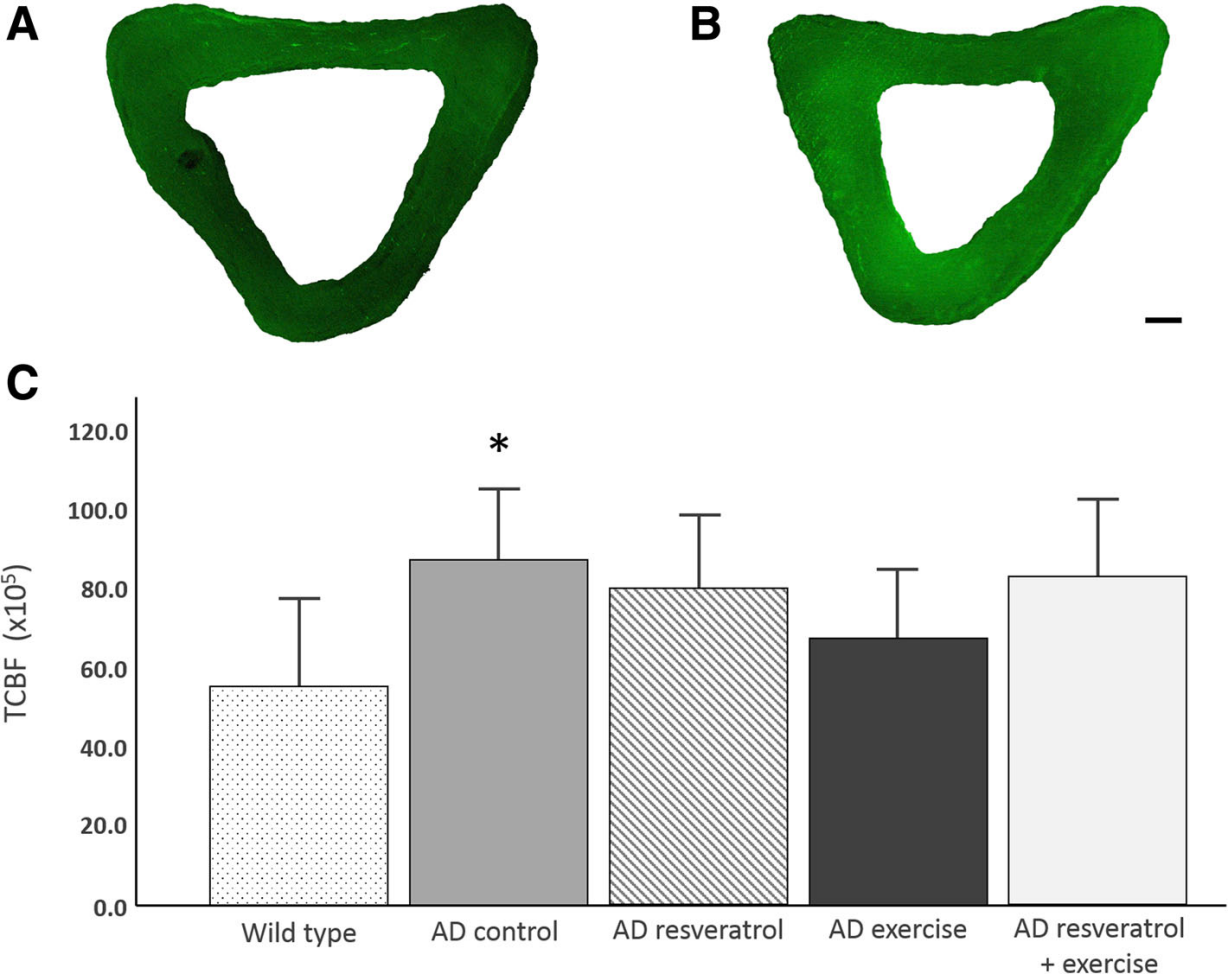
Comparison of corrected total bone fluorescence (CTBF) among treatment groups. **a** Tibia of a wild type mouse. **b** Tibia of an AD control mouse. **c** AD controls differed significantly from wild type mice (*P < 0.05). None of the AD treatment groups differed significantly from AD controls, nor did they differ significantly from wild type mice (*P* > 0.05 in both cases). Data are expressed as mean ± 2 SE. Scale bar 100 μm

Comparisons of CTBF show that tibias of 3xTg-AD mice have significantly greater AGE content than wild type mice (*P* < 0.05, Fig. 2). Treatment with resveratrol and exercise, either alone or in combination, did not result in significant differences with wild type or AD control mice (*P* > 0.05 in all cases). Wild type mice had the lowest CTBF while AD controls had the greatest CTBF on average.

## Discussion

The 3xTg-AD strain of mouse is triple transgenic, having transgenes for amyloid-β precursor protein, presenilin-1, and tau_P301L_. These mice exhibit signs of progressive cognitive and neuropathological deficits, and accumulation of amyloid-β and neurofibrillary tangles composed of hyperphosphorylated tau by six months of age [32, 33]. This model closely resembles the development and etiology of AD in humans. Patients with this debilitating disease also have an elevated fracture risk that is more than double the healthy population of the same age [9, 34]. To date, it is unknown if bone morphology and strength are compromised in 3xTg-AD mice. Here, we show 3xTg-AD mice tibia bones fracture under lower loads than age-matched, nontransgenic wild type counterparts. 3xTg-AD mice also display signs of reduced bone quality in comparison to wild type mice. Increased CTBF in 3xTg-AD mice indicates there is accelerated AGE accumulation in bone extracellular matrix, which has been directly linked to increased bone fragility and fracture risk [35–38]. Our findings suggest the decrease in bone quality may explain the increased fracture risk observed in 3xTg-AD mice more so than changes in bone morphology, as we find no difference in cross-sectional geometry between AD control and wild type mice. However, the cause of the increase in nonenzymatic glycation of bone proteins in 3xTg-AD mice is unknown. Evidence indicates that AGE accumulation contributes to amyloidosis in AD patients and such accumulations may be related to AGE increases in bone, but this hypothesis requires further testing [39].

Several investigations have focused on impeding the progression of AD-related cognitive deficits with exercise and resveratrol [13, 15, 40, 41]. These treatments are also known to have bone protective properties [42–44]. We applied both resveratrol and exercise training to the 3xTg-AD mice for a period of four months. Our results indicate that treatment with exercise and resveratrol, either alone and in combination, increase the maximum amount of force required to fracture tibias in comparison to AD controls. 3xTg-AD mice treated with these modalities have three-point bending test results within the range of those of wild type mice. 3xTg-AD mice treated with resveratrol and exercise also had increased bone diameter and total and cortical cross-sectional areas in comparison with untreated 3xTg-AD mice. Resveratrol has been shown to increase alkaline phosphatase activity, calcium deposition, and expression of anti-apoptotic and osteogenic regulatory proteins and transcription factors (osteocalcin, Osterix, Runx2/Cbfa1, Wnt, Sirt1) [45–47]. These stimulatory effects likely explicate the observed increases in bone diameter, area, and fluorescence, which contribute to the improved fracture resistance in these mice.

While we did not directly assess biochemical markers of bone quality, we measured CTBF, which reflects post-translational modification of structural collagen type I through nonenzymatic glycation. 3xTg-AD mice treated with resveratrol and exercise have CTBF values similar to wild type mice, suggesting treatment yields improvements in the quality of bone matrix. This finding is likely an effect of resveratrol treatment, which has been shown to inhibit the formation of AGEs, as well as stimulate expression of collage type I by osteoblasts [17, 48–50]. Of the two treatments in our study, resveratrol yields the greatest improvements in elastic modulus and stiffness, both of which are greatly affected by AGEs and are significant contributors to bone toughness [51]. Exercise treatment has also been shown to reduce AGE formation, although the mechanism is not as well understood [52]. Our results suggest actions of resveratrol and exercise may improve bone quality in 3xTg-AD mice, thereby improving fracture resistance.

The greatest improvement in three-point bending test results and cross-sectional geometric indicators of bone strength are observed in 3xTg-AD mice treated with exercise and resveratrol treatment. In combination, these therapies improve fracture resistance and bone strength to levels that are greater than those of untreated wild type mice. Prior studies have linked resveratrol treatment to improved aerobic performance and prevention of oxidative stress [53, 54] and increases in the activity of antioxidant enzyme systems [55]. It may be that resveratrol augments the beneficial effects of exercise to levels above those observed with each treatment in isolation. These results indicate exercise and resveratrol together may have bone-protective benefits beyond just the AD condition, but to the broader population.

## Conclusions

The evidence presented above suggests resveratrol and exercise may have therapeutic benefits for AD-related fracture risk. Exercise and resveratrol treatment may play a role in inhibiting the accumulation of AGEs in bone extracellular matrix to enhance bone strength and resistance to fracture.

## Abbreviations

AD: Alzheimer’s disease
AGE: Advanced glycation end products
Ct.Ar: Cortical area
CTBF: Corrected total bone fluorescence
Imax: Maximum second moment of area
Imin: Minimum second moment of area
J: Polar moment of area
M.Ar: Medullary area
Tt.Ar: Total area
